# Oribatid mites reveal that competition for resources and trophic structure combine to regulate the assembly of diverse soil animal communities

**DOI:** 10.1002/ece3.5409

**Published:** 2019-07-04

**Authors:** Matthew Magilton, Mark Maraun, Mark Emmerson, Tancredi Caruso

**Affiliations:** ^1^ School of Biological Sciences and Institute for Global Food Security Queen's University of Belfast Belfast UK; ^2^ JFB Institute of Zoology and Anthropology Georg August University Göttingen Göttingen Germany

**Keywords:** community assembly processes, competition, oribatid mites, soil animals, species co‐occurrence, stable isotope analysis

## Abstract

1. The role of niche partitioning in structuring species‐rich soil animal communities has been debated for decades and generated the “enigma of soil animal diversity.” More recently, resource‐based niche partitioning has been hypothesized to play a very limited role in the assembly of soil animal communities. To test this hypothesis, we applied a novel combination of stable isotopes and null models of species co‐occurrence to quantify the extent of resource niche partitioning on a diverse oribatid mite community sampled from mature oak woodland.
2. We asked whether species aggregate or segregate spatially and how these patterns correlated with the abundance of estimated trophic guilds. We also estimated the effects of environmental variables on community structure.
3. All measured environmental variables accounted for 12% of variance in community structure, including 8% of pure spatial structure unrelated to measured environmental factors and 2% of pure environmental variance unrelated to spatial variation. Co‐occurrence analysis revealed 10 pairs of species that aggregated and six pairs of species that were spatially segregated. Values of δ^15^N indicated that five out of the 10 pairs of aggregated species occupied the same trophic guild, while values of δ^13^C indicated that species in these five pairs consumed resources of different quality, supporting a significant role of resource‐based niche partitioning. Also, one of the five pairs of segregated species occupied the same trophic guild but had overlapping δ^13^C values suggesting that these species do not co‐occur locally and thus minimize competition for shared resources.
4. Partitioning of resources plays an underestimated role in soil microarthropod communities and different local communities consisted of the same trophic guilds with species identity changing from place to place. The sum of resource partitioning, multi‐trophic interactions, and microscale environmental variability in the environment is a viable solution to the enigma of soil animal diversity.

## 1 INTRODUCTION

Temperate forest soils support a vast diversity of soil fauna spanning many taxa. Together with microbes, this diversity of fauna forms complex food webs, which in turn underpin much of terrestrial ecosystem functioning. However, the cryptic and heterogeneous nature of soils makes it challenging to unravel the underlying ecological processes responsible for assembling and regulating these communities. Some progress has, however, been made in the last decade. Recently, ecologists (Davison et al., 2016; Götzenberger et al., 2012; Ingimarsdóttir et al., 2012; Nemergut et al., 2013) have focused on defining and quantifying the roles of various processes such as environmental filtering (Laliberte, Zemunik, & Turner, 2014), dispersal (Padial et al., 2014), and competition (Aerts, 1999). The majority of research on assembly processes has, however, focused on aboveground communities, notably plants (Cingolani, Cabido, Gurvich, Renison, & Díaz, 2007; Götzenberger et al., 2012; HilleRisLambers, Adler, Harpole, Levine, & Mayfield, 2012; Lambers, Clark, & Beckage, 2002; Mason, de Bello, Doležal, & Lepš, 2011; Spasojevic & Suding, 2012) and vertebrates (Andreassen, Stenseth, & Ims, 2002; Sutherland, Harestad, Price, & Lertzman, 2000). In soil communities, recent investigations have focused on assembly processes of bacteria (Nemergut et al., 2013) and fungi (Davison et al., 2016) with only a limited number of studies on soil microarthropods (Caruso, Taormina, & Migliorini, 2012; Lindo & Winchester, 2009; Maaß, Maraun, Scheu, Rillig, & Caruso, 2015; Nielsen et al., 2010).

In soil animal communities, Anderson (1975) early suggested that trophic niche differentiation through partitioning of resources can explain the coexistence of high numbers of species at small spatial scales. However, more recently the role of resource‐based niche partitioning in soil animals has been reconsidered, incorporating effects of trophic interactions and environmental filtering and downplaying the role of niche partitioning in soil animals (Maaß et al., 2015; Wardle, 2006). Further research has tested Anderson's hypothesis on oribatid communities using stable isotope methodology based on natural variations in ^15^N/^14^N to estimate trophic position (Schneider et al., 2004) and variation in ^13^C/^12^C to estimate consumption of different basal food resources between species (Pollierer, Langel, Scheu, & Maraun, 2009). Results indicated that in very diverse and phylogenetically old groups such as oribatid mites, species in the same assemblage span multiple trophic guilds, including phytophagous species (lichen, moss, and algal feeders), primary decomposers (detritivorous feeders), secondary decomposers (detritivorous/fungal feeders) and predators, scavengers and omnivores that feed on animal and fungal biomass (Maraun et al., 2011; Scheu & Falca, 2000; Schneider et al., 2004).

Although stable isotopes cannot identify the exact food source of a species, they can reveal relative differences between species in the isotopic space and map these differences onto relative differences in the trophic position of species, regardless of the type of food. Also, stable isotope information can be integrated with independent observations on mouth parts and direct feeding, which have so far confirmed the conclusions from stable isotope studies of soil fauna (Maraun et al., 2011; Perdomo, Evans, Maraun, Sunnucks, & Thompson, 2012; Schneider et al., 2004). Current evidence suggests that trophic niche differentiation through resource partitioning may indeed be an important underlying factor in assembling and regulating diverse mite communities in soil, and the results may well apply to other major taxa such as collembolans (Chahartaghi, Langel, Scheu, & Ruess, 2005; Maraun et al., 2011; Schneider et al., 2004).

Of all soil microarthropod groups, oribatid mites are the most diverse with currently around 10,000 described species (Norton, Behan‐Pelletier, Krantz, & Walter, 2009; Subías, 2004) and a total number of species estimated to be as high as 100,000, most of which inhabit soil (Schatz, 2002). Oribatid mites are also highly abundant with up to 200,000 individuals recorded per m^2^ in forest soils in temperate regions (Maraun & Scheu, 2000; Petersen & Luxton, 1982). Besides their diversity and abundance, oribatids provide and regulate important ecosystem functions including organic matter decomposition, both directly through consuming organic material (Pande & Berthet, 1973) and indirectly through regulation of fungal microbial communities via grazing (Moore, Walter, & Hunt, 1988), and nutrient cycling through digesting leaf litter and excreting fecal matter (Swift, Heal, & Anderson, 1979; Wardle, 2006). This combination of high diversity, high abundance, and both direct and indirect links to critical ecosystem processes makes oribatid mites an interesting model group for investigating the processes that assemble and regulate biological communities.

To investigate the significance of resource niche partitioning, we used a novel combination of natural variations in ^15^N/^14^N and ^13^C/^12^C stable isotope and species co‐occurrence analysis. We tested whether species aggregate or segregate spatially based on trophic guild (estimated by nitrogen signatures) and/or resource overlap (estimated by carbon values). Therefore, if resource partitioning does play a role in assembling and regulating oribatid mite communities, we hypothesize that aggregating species will occupy a different trophic guild and/or consume different resources while species consuming very similar resources should be segregating spatially to minimize competition. If trophic position does not contribute to patterns of species co‐occurrence, we hypothesize a general lack of correlation between patterns of segregation and aggregation and stable isotope values.

The key hypotheses of this work revolve around detecting systematic relationships between species co‐occurrence and trophic position in the local communities. More specifically, we tested two hypotheses: (a) co‐occurring species within the same trophic guild (overlapping values of δ^15^N) reduce competition via trophic differentiation (not overlapping values of δ^13^C) or they segregate spatially in different local assemblages to minimize competition and (b) species assemblages of oribatid mites are organized in multiple trophic guilds consistently across multiple sites, which reduces competition for the same resources and should result in nonoverlapping values of δ^15^N for aggregating species.

## 2 MATERIALS AND METHODOLOGY

### 2.1 Study site

This study was conducted in Breen Oak Woodland, Armoy, Northern Ireland (N55°08.510 W006°14.807). The forest covers an area of 15.5 hectares with *Quercus petraea*—(Sessile Oak) as the dominant species. Other species present include *Alnus glutinosa*—(Alder), *Corylus avellana*—(Hazel), *Ilex aquifolium*—(Holly), *Sorbus aucuparia*—(Rowan), *Pinus sylvestris*—(Scots Pine), and *Betula pendula*—(Silver Birch). Understorey vegetation is dominated by *Luzula sylvatica*—(Great Wood‐Rush) with patches of mixed grass species scattered throughout. The forest is situated on steep rolling topography with acidic, nutrient poor soils ranging from clay to sandy loams. Soil types include podzols situated on ridge tops, brown podzolic intermediate soils on the slopes, and brown/young earth soils on the valley base.

### 2.2 Experimental design

Ten plots (each 2 m^2^) were established within a 600 m × 400 m study area to sample small‐scale species assemblages in 60 local spots. Per plot, six soil cores (10 cm in diameter and 5 cm in depth) were collected to represent local species assemblages at a small spatial scale, which is appropriate for these animals given their body size and dispersal abilities (Caruso et al., 2012; Lindo & Winchester, 2009; Maaß et al., 2015; Nielsen et al., 2010). Cores were collected randomly within each plot, and the position of each sample within each plot was recorded, via measuring the distance (cm) from the GPS geo‐referenced North‐East corner of each plot using a compass and rulers, and then converted into UTM coordinates resulting in a final estimated accuracy of ±5 cm. A small subsample (approx. 10 g) was extracted from each core and used to measure soil water content and pH.

Given the small spatial scale of the study area, each of the 10 defined plots was selected at a minimum distance of 15 m apart to maximize the range of variation in key environmental factors and oribatid mite diversity. The small scale of the study is suitable to detect spatial and resource niche partitioning between species inhabiting different local spots within and between plots and within the whole single oribatid community of the forest. The measured environmental factors included vegetation composition, natural litter density, elevation, and the spatial position of each sampled spot. In order to simplify the experimental design while maximizing the range of environmental heterogeneity, understory vegetation composition was defined as either dominated by *Luzula sylvatica* or grass spp. Also, litter density was defined as high (>500 g d.w. per m^2^) and low (<40 g d.w. per m^2^), with values of dry biomass based on prior field estimates (not shown). To represent observed percentage cover of understory vegetation and natural litter densities throughout the study area, we were able to identify six plots fully dominated (cover > 90%) by *L. sylvatica* (three containing high litter density and three containing low litter density) and four plots dominated by grass spp. (two containing high litter density and two containing low litter density). Ideally, we would have used a balanced design; however, we were constricted by the availability of suitable plots representing > 90% grass cover. Although different plant species may result in variation in resource inputs, this sampling factor was of minor importance to test our hypotheses, which mainly focused on local species assemblages. Instead, we used plots and the two main types of plant cover observed in the forest just to maximize the range of soil moisture and soil pH. Values for both moisture and pH were obtained for every sample and then averaged to gain a single value per plot (see: Table S3). Also, we used spatially explicit analyses (see below) that directly accounted for autocorrelation in species distribution between samples (the 60 corers), and each soil sample could then be formally treated as an independent replicate collected within each plot (see below for statistical methods). With this design, we aimed to detect how species can partition space and potentially resource in a relatively homogenous area while accounting for environmental variation within the area.

Soil fauna were extracted using Tullgren funnels (Tullgren, 1918) with a 2‐mm mesh for a period of 7 days and preserved in 75% ethanol for identification. Oribatid mites were separated from all other fauna and identified to species level using (Weigmann, 2006) and species distributions and reviews cited therein.

### 2.3 Species distribution and stable isotope data

All species were identified as either present or absent in all cores. These data were compiled to create a species presence/absence matrix for analysis of species co‐occurrence. For stable isotope analysis, multiple individuals of each species were transferred to tin capsules and weighed. To reach the required mass for accurate analysis, between 1 and 50 individuals were used per capsule depending on relative body size of the species being measured. Cryptic species, that is, *Suctobelbella* spp., were pooled and analyzed at the genus level. Samples were dried at 60°C for a minimum of 12 hr, reweighed, and placed in a desiccator awaiting further analysis. Both litter and soil samples were mixed to create a composite sample, ground, and prepared using the same methods as above. Measured composite litter/soil samples served as a baseline of δ^15^N and δ^13^C values against which oribatid N and C values were calibrated. Oribatid trophic guilds/basal resources were defined based on the assumptions that each trophic guild spans approximately 3.4‰ for nitrogen ratios and a change of approximately 1.0‰ in carbon represents a change in basal food resources (Post, 2002).

The values of ^15^N/^14^N and ^13^C/^12^C ratios were measured using a combined system of a mass spectrometer (Delta V Plus Thermo Electron) and an elemental analyzer (Euro EA 3000, Euro Vector S.p.A.) after Reineking, Langel, and Schikowski (1993). Atmospheric nitrogen was used as the standard for ^15^N calibration, Vienna Pee Dee Belemnite (V‐PDB) for ^13^C calibration: see (Schneider et al., 2004) for more details, and acetanilide (C_8_H_9_NO) was used for internal machine calibration.

To account for possible intraspecific variation of isotopic ratios within species, on average 32 individuals (extracted from across 10 soil cores, when possible) were used for isotopic analysis: with a maximum of 50 individuals used in a single replicate measurement (for small‐bodied species). A single isotopic value was obtained via averaging six replicate measurements completed in the following format: two replicates of a single individual, two replicates of five individuals, and two replicates of 10 individuals (or up to 50 specimens for small‐bodied species).

### 2.4 Statistical analysis

Mean and standard errors of measured isotopic ratios were calculated and plotted to visualize estimated trophic position of all species relative to the measured composite litter/soil baseline. Species were assigned to trophic guilds based on their respective isotopic values from this study, findings from previous studies describing trophic position, and the morphology of feeding mouth parts (Maraun et al., 2011; Perdomo et al., 2012).

Multivariate patterns in species distribution were analyzed using principal coordinates analysis on the Jaccard distance matrix and so distance‐based redundancy analysis (RDA) to quantify the effects of environmental variables on species distribution (Legendre & Gallagher, 2001; Legendre & Legendre, 1998). We also used principal coordinate analysis of neighborhood matrices (PCNM; see: Borcard, Legendre, Avois‐Jacquet, & Tuomisto, 2004 for further details) to account for spatial autocorrelation at multiple spatial scales. Each PCNM eigenvector describes autocorrelation at a specific spatial scale (e.g., within plot). These eigenvectors thus quantify spatial patterns in the multivariate species distribution, and these patterns are due to a combination of factors, many of which are often not measured (e.g., clustering due to intraspecific interactions). The set of eigenvectors are often called “spatial factors” or “space” and are used in statistical inference to remove autocorrelation and variation that is not attributable to measured covariates (e.g., pH). Following Dray, Legendre, and Peres‐Neto (2006), we used the AIC criterion to select a subset of parsimonious eigenvectors which accounted for the largest possible amount of variation within the species matrix. Variance partitioning was calculated to quantify the amount of variation accounted for by environmental variables, spatial eigenvectors, and the variance shared between environment and spatial eigenvectors. Multivariate analyses were completed in R version 3.4.3 (R Core Team, 2017) using the package vegan (Oksanen et al., 2013).

To investigate if oribatid species distribute spatially according to their ^14^N/^15^N and ^12^C/^13^C isotope values, the original species presence/absence matrix was reformatted to include only species that had been characterized isotopically (25 species). We used the C‐score to quantify patterns of co‐occurrence. The index quantifies checkerboard distributions so that species that do not co‐occur very often produce a high index value and vice versa. High value of the index thus means spatial segregation and vice versa (Stone & Roberts, 1990). We applied null model analysis (Gotelli, 2000) to the C‐score preserving row and column totals (Gotelli, 2000). This approach is ideal to test for nonrandom patterns due to species interactions because it affects only species composition. The combination of C‐score and preservation of row and column totals has been shown to have very good statistical properties and minimize the risk of false positives (Gotelli, 2000). The null distribution of the C‐score was obtained from 5,000 random matrices. The central tendency of the null distribution was then compared to the observed C‐score. The C‐score was also calculated on a species‐pair basis and tested following the method of (Gotelli & Ulrich, 2010) and the Fortran program Pairs (Ulrich, 2008): this method builds confidence limits using the empirical Bayes approach. Effect size was calculated as $\frac{\text{obs}.\text{index}-\exp.\text{index}}{\text{null}\;S.D.}$, where obs.index is the observed C‐score, exp.index is the central tendency in the C‐score null distribution, and null S.D. is the standard deviation of the C‐score null distribution. Significant pairs were extracted from the model output and directly compared to their corresponding assigned trophic guilds (defined via their respective nitrogen isotopic values) and relative positions within those guilds. See also Caruso, Hogg, et al., (2019); Caruso for further details on the null model methods.

## 3 RESULTS

### 3.1 Fauna

**Figure 1:**
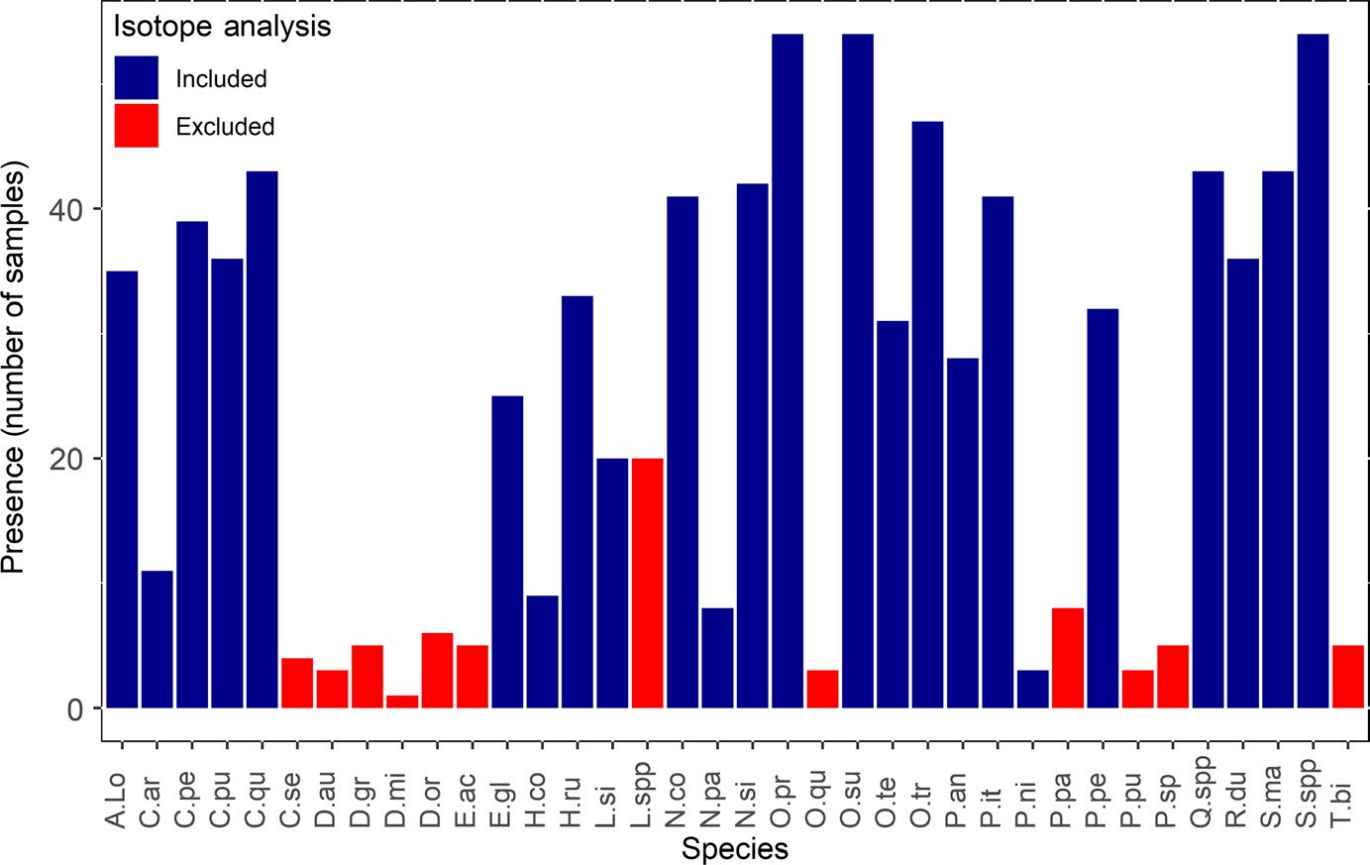
Number of times each recorded species was present in a single sample from all 60 samples. Blue and red bars represent species included and excluded from stable isotope analysis, respectively

A total of 37 species were found in the study. The most frequent (>20 samples; Figure 1) species were *Ceratozetes peritus*, *Ceratoppia quadridentata*, *Nanhermannia coronata*, *Nothrus silvestris*, *Oppiella propinqua*, *Oppiella (R.) subpectinata*, *Oppiella (M.) translamellata*, *Phthiracarus italicus*, *Quadroppia* spp. *Steganacarus magnus,* and *Suctobelbella* spp. These species are all mesophilous species, very typical of temperate broadleaved forests. Sample species richness ranged from 24 to 3, with no relation to understory vegetation and litter density, and on average, there were 15 species per sample (Table S1), with turnover in sample species composition within each plot. Twelve species were excluded from isotopic analysis because they were either too rare or had insufficient biomass for isotopic analysis.

### 3.2 Effects of environmental variables

**Figure 2:**
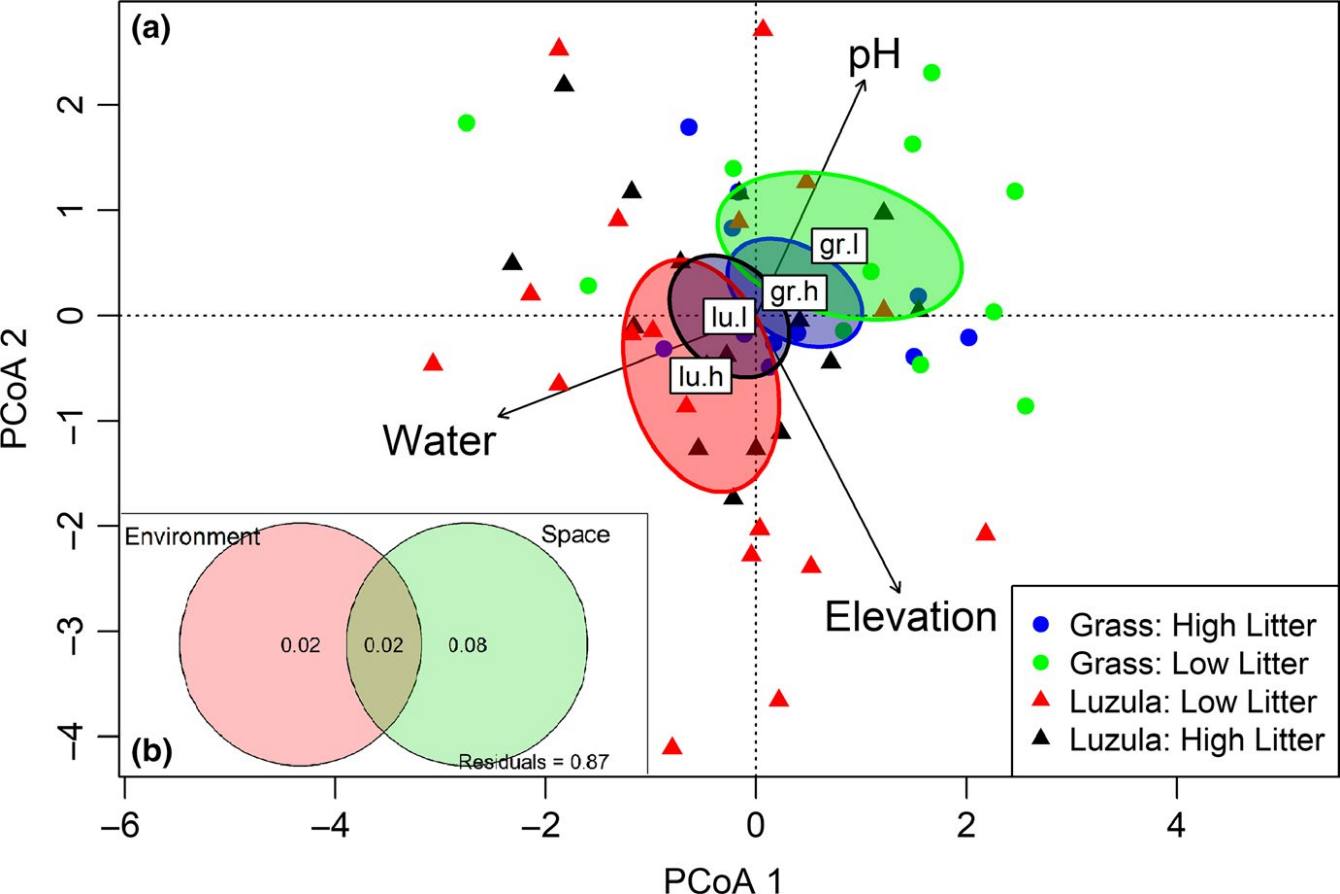
(a) PCoA analysis of all measured environmental factors and their relative importance in driving oribatid mite community structure. Gr.l: Grass, low litter density, gr.h: Grass, high litter density, lu.l: Luzula, low litter density and lu.h: Luzula, high litter density. (b) Venn diagram illustrating percentage of community structure regulated by combined environmental factors, stochastic spatial variation, and their shared variation

Percentage water content ranged from 10.8% to 79.6% while pH ranged from 3.12 to 5.34, indicating a good range of environmental variation that could structure the community. Redundancy analysis (Figure 2a) and variance partitioning (Figure 2b) indicated that 4% of community structure was attributable to measured environmental variables. Only 2%, however, was uniquely attributable to these environmental variables after removing spatial autocorrelation. This fraction was statistically significant at a *p* < 0.05. Also, 8% of variation was accounted for just by PCNM spatial eigenvector, independently of environmental factors. Residuals summed up to 87% of variance in community structure and total variance explainable by measured environmental variation and spatial autocorrelation equals 12%. Each environmental correlate of oribatid community structure (Figure 2a) was individually tested for statistical significance using a permutational approach and only percentage water content was significant (*p*‐value = 0.028).

### 3.3 Stable isotopes and inference of trophic structure

**Figure 3:**
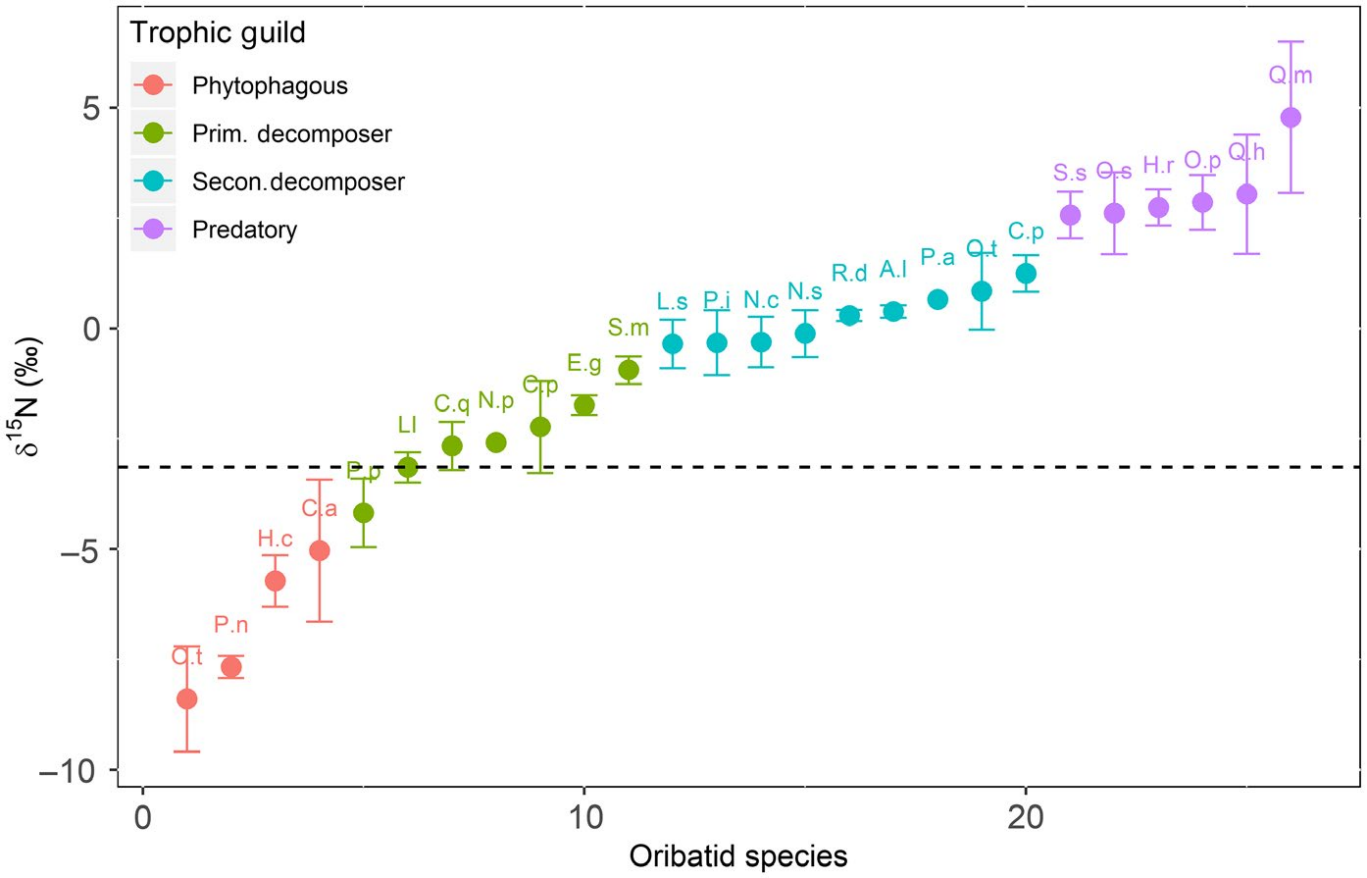
Measured δ^15^N isotopic values for 25 oribatid mite taxa (24 species and one genus). Red, green, blue, and purple represents phytophagous/lichen feeder, primary decomposer, secondary decomposer and top level (predators/scavengers) trophic guilds, respectively. Dashed line indicates isotopic baseline (composite litter/soil samples) used to assign trophic guilds. Standard error bars represent average variation in δ^15^N measurements between replicate samples

A total of 25 species and composite (litter/soil) samples were subjected to isotopic analysis. Measured δ^15^N values of combined litter/soil averaged −3.14‰. Oribatid mite species δ^15^N values (Figure 3) spanned a range of 11.53 δ units, from −8.39‰ (*Ophidiotrichus tectus*) to 4.78‰ (*Quadroppia monstruosa*). Based on the assumption of a δ^15^N enrichment of approximately 3.4‰ per trophic guild (Minagawa & Wada, 1984; Post, 2002), on a baseline value of −3.14‰ and also based on the morphology of oribatid species feeding mouth parts (chelicera) as a guide (Wallwork, 1958; also see: Table S2 for chelicera), the measured δ^15^N values suggested four trophic guilds that were identifiable in the present study (Figure 3) and were defined as follows: (a) phytophagous species: from −8.39‰ (*O. tectus*) to −5.03‰ (*Carabodes areolatus*), (b) primary decomposers: from −4.18‰ (*Platynothrus peltifer*) to −0.94‰ (*S. magnus*), (c) secondary decomposers: from −0.35‰ (*Liebstadia similis*) to 1.24‰ (*C. peritus*), and (d) predatory: from 2.57‰ (*Suctobelbella* spp.) to 4.78‰ (*Q. monstruosa*).

**Figure 4:**
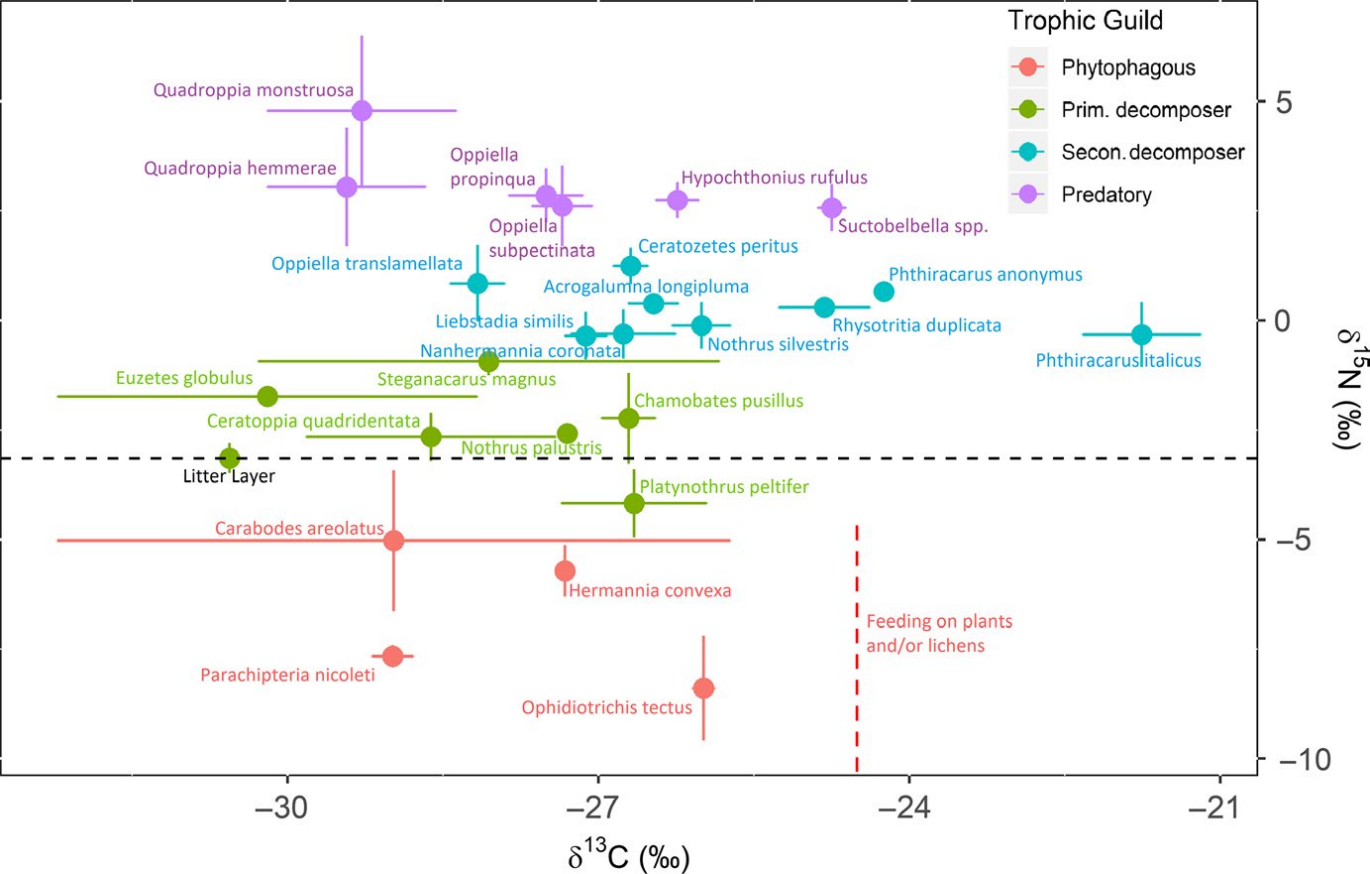
δ^15^N values and δ^13^C for 25 Oribatid taxa. Red, green, blue and purple represent Phytophagous (incl. lichen feeders), primary decomposers, secondary decomposers and top level (predators/scavengers) guilds, respectively. Dashed line represents isotopic baseline (composite litter/soil samples) used to calibrate trophic guilds. Standard error bars show variation in δ^15^N and δ^13^C between replicate samples

In addition to δ^15^N values, δ^13^C values were also measured for all species, with the exception of *Neoconocephalus palustris* and *Phthiracarus anonymus* due to insufficient biomass required for accurate measurements. Both δ^15^N and δ^13^C values were combined and plotted to investigate the isotopic structure of the oribatid mite community (Figure 4; see also Tables S2 and S3). The δ^13^C values of combined litter/soil averaged −30.56‰. δ^13^C values for oribatid taxa spanned a range of 8.43 δ units, from −30.19‰ (*Euzetes globulus*) to −21.76‰ (*P. italicus*). Trophic guilds, defined by δ^15^N values, also spanned a range of δ^13^C values: (a) phytophagous species: from −28.98‰ (*Parastacus nicoleti*) to −25.99‰ (*O. tectus*), (b) primary decomposers: from −30.19‰ (*E. globulus*) to −26.66‰ (*P. peltifer*), (c) secondary decomposers: from −28.17‰ (*O. (M.) translamellata*) to −21.76‰ (*P. italicus*), and (d) predatory species: from −29.43‰ (*Quadroppia hammerae*) to −24.75‰ (*Suctobelbella* spp.).

### 3.4 Patterns of species co‐occurrence

Based on the 25 oribatid taxa for which stable isotope analysis was undertaken, null model co‐occurrence analysis found 16 statistically significant pairs of species, of which 10 were pairs of aggregating species and six were pairs of segregating species. Co‐occurrence data for significant species pairs were combined with their respective isotopic signatures to investigate whether species distribution was regulated by species trophic position (Table 1). Of the 10 aggregating pairs, six represented species occupying the same trophic guild: three in the predatory guild, one in the secondary decomposer guild, and two in the primary decomposer guild. The remaining four pairs represented species occupying different trophic guilds. Of the six segregating species pairs, two represented species sharing the same trophic guild, both occupying the secondary decomposer compartment, and four represented species occupying different trophic guilds.

**Table 1:** Combined results of co‐occurrence and stable isotope analysis

Species pair		Body size (µm)		Guild		Z‐Score	P‐value	Spt. pat.	Isotopic ratio overlap between species pairs	
Spp. A	Spp. B	Spp. A	Spp. B	Spp. A	Spp. B				^15^N/^14^N	^13^C/^12^C
*Oppiella propinqua*	*Suctobelba spp.*	265–315	160–300	1	1	−2.84	0.004	Agg.	Yes	No
*Oppiella propinqua*	*Quadroppia spp.*	265–315	155–230	1	1	−2.00	0.045	Agg.	Yes	No
*Suctobelba spp.*	*Quadroppia spp.*	160–300	155–230	1	1	−2.22	0.026	Agg.	Yes	No
*Ceratozetes peritus*	*Oppiella translamellata*	380–400	260–350	2	2	−2.16	0.030	Agg.	Yes	No
*Nanhermannia coronata*	*Acrogalumna longipluma*	480–570	625–790	2	2	2.53	0.011	Seg.	Yes	Yes
*Oppiella translamellata*	*Nanhermannia coronata*	260–350	480–570	2	2	2.22	0.026	Seg.	Yes	No
*Chamobates pusillus*	*Ceratoppia quadridentata*	370–470	500–600	3	3	−2.26	0.023	Agg.	Yes	No
*Steganacarus magnus*	*Platynothrus peltifer*	700–1200	770–980	3	3	−2.12	0.034	Agg.	No	Yes

*Ceratozetes peritus*	*Quadroppia spp.*	380–400	155–230	2	1	−2.16	0.031	Agg.	No	No
*Nanhermannia coronata*	*Hypochthonius rufulus*	480–570	650–700	2	1	2.22	0.026	Seg.	No	Yes
*Nanhermannia coronata*	*Parachipteria nicoleti*	480–570	550–700	2	4	2.24	0.025	Seg.	No	No
*Nothrus silvestris*	*Ophidiotrichus tectus*	710–810	240–270	2	4	1.96	0.050	Seg.	No	Yes
*Nothrus silvestris*	*Hyptiocheta convexa*	710–810	1170–1520	2	4	2.51	0.012	Seg.	No	No
*P. italicus*	*Ophidiotrichus tectus*	510–670	240–270	2	4	−2.00	0.046	Agg.	No	No
*Ceratoppia quadridentata*	*Liebstadia similis*	500–600	500–600	3	2	−2.02	0.043	Agg.	No	No
*Steganacarus magnus*	*Acrogalumna longipluma*	700–1200	625–790	3	2	−1.99	0.047	Agg.	No	Yes

Note

Table illustrates significant species co‐occurrence combinations, body size combinations (µm), and their respective trophic guilds. Z‐score used as an estimate of species aggregation or segregation. Spt. Pat.—Spatial Pattern. Isotopic ratio overlap: indicated whether species within pairs overlapped in their respective ^15^N/^14^N and ^13^C/^12^C values, respectively. Gray bar represents a division between species within pairs belonging to occupying the same or different trophic guilds. For full species names, see Table S2.

## 4 DISCUSSION

### 4.1 Trophic structure and resource partitioning

In aboveground systems, reduced competition via resource partitioning plays a major role in driving species diversity and composition (HilleRisLambers et al., 2012; Schoener, 1974). Anderson (1975) early suggested niche partitioning via trophic differentiation may partially explain the coexistence of large numbers of species at small spatial scale in soil. Recent investigations (Corral‐Hernández, Maraun, & Iturrondobeitia, 2015; Pollierer et al., 2009; Scheu & Falca, 2000; Schneider et al., 2004) have explored this hypothesis using stable isotope analysis as an indirect way to estimate both the number of trophic guilds present within a community and which species occupy these guilds. In our study, a total of 603 individuals representing 25 species were subjected to isotopic analysis. Final isotopic values of each species were an average of six replicate measurements that showed variation in isotopic values within species. This variation, which we could not resolve in this study, is likely due to natural plasticity in species diet and the fact that different individuals and populations of the very same species may access different resources in different places, depending on resource distribution and availability (Schneider et al., 2004). This implies a degree of trophic generalism, and in the following discussion, we show multiple lines of evidence for this.

We assumed a change in nitrogen isotopic values of approximately 3.4‰ per trophic guild and a 1‰ change in carbon isotopic ratios representing a change in food resources (Post, 2002). With this assumptions, measured isotopic values from previous studies found evidence of 3–4 trophic guilds with δ^15^N values spanning over 12 δ units and δ^13^C values spanning 4 δ units (Pollierer et al., 2009; Schneider et al., 2004). Our results are consistent with this range of values as the total δ^15^N and δ^13^C isotopic values range was 11.53 δ units and 8.43 δ units, respectively. The comparison of δ^15^N and δ^13^C of individual species with composite litter/soil sample values and previous finding on the trophic structure of soil fauna communities, for example (Corral‐Hernández et al., 2015; Pollierer et al., 2009; Scheu & Falca, 2000; Schneider et al., 2004), and the morphology of feeding mouth parts (Perdomo et al., 2012) indicated that the oribatid mite community of this study consisted of at least four trophic guilds. There are phytophagous species, primary decomposers, secondary decomposers, and species feeding at a trophic level higher than the secondary decomposer guild (e.g., predators/scavengers). Clearly, species within different trophic guilds are very unlikely to compete for resources, which already explains the coexistence of many oribatid species at the very local scale of a single soil sample. This can also partly explain why the measured environmental variables accounted for a small fraction of community variance, a result that is consistent with previous studies (Maaß et al., 2015). Variance partitioning also showed that 8% of oribatid community structure was spatially structured but not explainable by the measured environmental variables. This variation can be due to a combination of unmeasured environmental variation, dispersal limitation, and other unmeasured population factors that operate at scales smaller than those accounted for in our study (Lindo & Winchester, 2009). Furthermore, residuals indicated an overwhelming 87% of community structure remained unexplained, indicating that the measured environmental variables across the spatial extent of our study site are a poor predictor of community structure. This is not unusual at the spatial scale of this investigation (Maaß et al., 2015) and potentially suggests an important role of stochastic factors in the assembly of these communities (Caruso et al., 2012; Maaß et al., 2015). This role usually decreases with the increase in the spatial extent of studies (Caruso, Hogg, et al., 2019; Caruso, Schaefer, et al., 2019; Zaitsev, Straalen, & Berg, 2013).

Despite the observation that species are arranged into discrete trophic guilds, each guild consists of multiple species, which could still compete for resources. Phytophagous feeding species (Chahartaghi et al., 2005), which feed on algae and/or lichens, included four species (*O. tectus*, *P. nicoleti*, *Hyptiocheta convexa*, and *C. areolatus*) with δ^13^C values spanning 2.99 δ units. Given there was no overlap in ^12^C/^13^C between *O. tectus, P. nicoleti,* and *H.* *convexa*, these species may specialize on different primary food resources. However, these three species co‐occurred randomly with respect to each other. *C. areolatus* showed overlap with all other phytophagous feeding species and also co‐occurred randomly with them, indicating that this species may utilize multiple resources, thus being a generalist.

Species assigned to the primary decomposer trophic guild have δ^15^N values similar to the composite litter/soil values (−3.14‰) spanning a range of 3.24 δ units. The primary decomposers included *P. peltifer*, *C. quadridentata*, *N. palustris*, *Crypturgus pusillus*, *E. globulus*, and *S. magnus*. Similar δ^15^N values have been reported in other studies for *P. peltifer*, *S. magnus* (Corral‐Hernández et al., 2015; Pollierer et al., 2009; Scheu & Falca, 2000; Schneider et al., 2004), *E. globulus* (Pollierer et al., 2009; Schneider et al., 2004), and *N. palustris* (Schneider et al., 2004). The literature shows mixed results for *Chamobates* species but our results classified the species *C. pusillus* as a primary decomposer. Schneider et al. (2004) recorded this species as a secondary decomposer, and Heidemann, Scheu, Ruess, and Maraun (2011) found evidence of some *Chamobates* species consuming nematodes suggesting it may be omnivorous. Overall, these results suggest that while some *Chamobates* species might be primary decomposers, other species in the genus are capable of feeding at higher trophic levels, making the genus very heterogeneous and species within the genus potentially evolving different trophic strategies to partition resources. Also, the range of values observed for δ^13^C values (8.43 δ units in total) supports the idea that these species utilize multiple food resources, if we assume a change in basal food resources for every 1‰ increase in ^13^C δ units. Additionally, ^12^C/^13^C standard errors showed large variation with overlap between species, providing further evidence that primary decomposers are generalists rather than specialist feeders.

Our results also categorized *P. peltifer* and *S. magnus* as primary decomposers although other studies have reported these species also consumes nematodes (Heidemann et al., 2014, 2011) providing further evidence of a generalist feeding strategy. In this study, and in investigations conducted by others (Corral‐Hernández et al., 2015; Schneider et al., 2004), the secondary decomposer guild was the most diverse containing nine species: *L. similis*, *P. italicus*, *N. coronata*, *N. silvestris*, *Rhysotritia duplicata*, *Acrogalumna longiplumna*, *P. anonymous*, *O. (M.) translamellata,* and *C. peritus*. Schneider et al. (2004) classified *N. coronata* and *Chamobatidae* species as secondary decomposers but assigned *R. duplicata* and *Phthiracaridae* spp. to primary decomposers, with *N. silvestris* and *C. peritus* also being categorized as secondary decomposers by Scheu and Falca, (2000) and Corral‐Hernández et al., (2015), respectively. The classification of individual species into different trophic guilds shows how trophic behavior of species within oribatids is very heterogeneous with no perfectly discrete trophic levels. This might imply that the spatial scale at which resource partitioning can occur and allow coexistence of multiple species is more variable than in typical aboveground food webs. Wallwork (1958) also documented *P. italicus* feeding on woody tissue, suggesting this species may feed on both detritus and fungal species and thus potentially competes with species within two different trophic guilds. Our results suggest *N. silvestris* to be a secondary decomposer species. Schneider et al. (2004) classified this species as a predatory/scavenger species but Schneider and Maraun (2005) provided evidence that *N. silvestris* consumes a variety of ectomycorrhizal fungal species, and two other studies (Heidemann et al., 2014, 2011) documented that *N. silvestris* also consumes nematodes, making the species very generalistic. Our results classified *A. longiplumna* as a secondary decomposer. However, Schneider et al. (2004) categorized the related species *Galumna* spp. within the predatory trophic guild and Heidemann et al. (2014) showed evidence of *Galumna* spp. also consuming nematodes. Thus, wide niche breadth seems to exist between all the major trophic guilds with a number of genera, which have species that display very different strategies.

Species that might be either predators, scavengers, or omnivorous (i.e., feeding at the highest trophic level), included *Suctobelbella* spp., *O. propinqua*, *O. (R.) subpectinata*, *Hypochthonius rufulus*, *Q. hammerae* and *Q. monstruosa*. *Oppiidae* spp., *Suctobelbidae* spp. and *H. rufulus*. These species had the highest δ^15^N values of all, a result that is consistent with the investigation conducted by Schneider et al., (2004) who also defined *H. rufulus*, *Oppiidae*, and *Suctobelbidae* as predators, scavengers, or omnivorous feeders. Corral‐Hernández et al. (2015) also categorized *Oppiidae* spp. within the predatory feeding guild, and earlier authors (Rockett, 1980) reported that *Oppiidae* can feed directly on nematodes, which provides substantial and independent evidence of a predaceous feeding strategy at least for some species. Pollierer et al. (2009) classified *H. rufulus* within the predatory guild, while much earlier observations (Riha, 1951) observed this species feeding on dead collembolans, making *H. rufulus* a scavenger. Thus, once again, most evidence suggests that within the highest trophic levels there actually is a broad range of food resources, with each resource possibly being utilized by different species in response to competition for resources at local scales. For example, when we consider species within the same genus, δ^15^N values indicate possible signs of resource partitioning. *Oppiella* species span a range of 2.01‰ across two trophic guilds with both *O. propinqua* and *O. (R.) subpectinata* occupying the predatory guild (2.85‰ and 2.61‰ nitrogen, respectively) and *O. (M.) translamellata* found in the secondary decomposer guild (0.84‰). *Nothrus* species span a range of 2.47‰ across two trophic guilds with *N. silvestris* occupying the secondary decomposer guild (−0.11‰ nitrogen) and *N. palustris* located in the primary decomposer compartment (−2.58‰ nitrogen), results all consistent with Schneider et al. (2004).

All these results provide very robust evidence that oribatid species assemblages are very structured from a trophic point of view, both in terms of number of trophic guilds and potential partitioning of resources within guilds and also with many species showing much potential for being very generalist in their diet.

### 4.2 Species co‐occurrence and resource partitioning

Isotopic characterization in combination with an analysis of species co‐occurrence patterns further supports a key role of resource partitioning through trophic differentiation at least for some pairs of species. For five out of the six pairs of species that aggregated significantly (i.e., found in the same sample more often than expected by chance) and belonged to the same trophic guilds, no overlap in δ^13^C values implies that species can coexist locally by accessing different items of food at the same trophic level. Alternatively, *A. longiplumna* and *N. coronata*, which belong to the same trophic guild, displayed small and overlapping variation in their δ^13^C value and also segregated significantly, which suggests that these species may compete in a way that limits their ability to share resources locally, that is, they experience strong competition with one another and thus live in different places.

Thus, for one‐third of the pairs of species that showed significant co‐occurrence patterns, stable isotopes highlighted that resource niche partitioning can play a major role in driving species distribution and composition. However, the remaining two‐thirds of significant co‐occurrence patterns could not be interpreted in terms of overlaps in the stable isotope space, suggesting that other factors determine these co‐occurrence patterns. Notably, all plots always contained all four trophic guilds (Table S3) with usually four and in most plots at least five species in three guilds and an average of three species in the remaining guild. This observation suggests a relatively stable trophic structure that seems independent of vegetation composition, litter density, water content, and pH. These findings also suggest that functional redundancy within trophic guilds is a consistent feature of oribatid mite communities simply because the same trophic guild is represented by multiple species in very local assemblages (cores or plot) but with species identity changing from place to place, which might also explain why we found that general environmental variables such as soil moisture, pH, and litter type affected community structure only to a very small extent at the spatial scale of our study. Our observations and inference of functional redundancy are likely to apply very generally to these communities if one retrospectively reconsider results from previous stable isotope analyses of other soil microarthropod communities (Pollierer et al., 2009; Schneider et al., 2004). Overall, the hypothesis of functional redundancy is also consistent with earlier findings for various groups of soil organisms including microbes (Mikola & Setälä, 1998) and collembolans (Cragg & Bardgett, 2001).

## 5 CONCLUSIONS

At the relatively small spatial scale of this study, a main factor structuring the investigated animal communities is the general trophic structure represented by the four major trophic guilds, which might indirectly reflect competition for resources in the past (e.g., species in the same genus that feed on different food) and, to a lesser extent, current resource niche partitioning that affects patterns of co‐occurrence between some species pairs. These two factors are likely to operate alongside the role of microscale environmental filtering, which may further support niche partitioning. Thus, overall our study resurrects Anderson's hypothesis (Anderson, 1975) that partitioning of resources within and between trophic guilds plays an underestimated role in structuring exceptionally species‐rich soil animal communities.

## CONFLICT OF INTEREST

None declared.

## AUTHOR CONTRIBUTIONS

MMg, ME, and TC conceived the study and set up the field plots. MMg and MM analyzed the samples. MMg and TC analyzed the data. All authors contributed to the interpretation of results and the write‐up of the ms and finally approved the ms.

## Data Availability

Upon acceptance, all data will be deposited on a publicly accessible repository such as Dryad. Part of the data area also available in the Supporting Information.
