# Supplementary material for: Oribatid mites reveal that competition for resources and trophic structure combine to regulate the assembly of diverse soil animal communities
Source: Ecol Evol. 2019 Jul 4;9(14):8320–30. doi: 10.1002/ece3.5409 (PMC6662270; doi:10.1002/ece3.5409)
Supplement: Supplementary file 1 [file ECE3-9-8320-s001.docx]

**Table S1.** Maximum, average and minimum number of species found in a single sample, vegetation type (*Luzula* and grass spp.) and leaf litter (High Lit. Den. and Low Lit. Den.).

| **Species No.** | **Sample** | **Luzula spp.** | **Grass spp.** | **High Lit. Den.** | **Low Lit. Den.** |
| --- | --- | --- | --- | --- | --- |
| Maximum | 24 | 24 | 24 | 24 | 24 |
| Average | 14.6 | 13.7 | 15.8 | 14.7 | 14.4 |
| Minimum | 3 | 4 | 3 | 6 | 3 |

**Table S2** Systematic representation of oribatid taxa feeding guilds present in Breen Oak Woodland. NA represents rare and not abundant species that could not be analysed for stable isotopes, Trophic guilds were defined by the following δ^15^N signatures: - 1: -8.39‰ to -4.18‰, 2: -4.18‰ to -0.35‰, 3: -0.35‰ to 2.57‰ and 4: 2.57‰ to 4.78‰. Chelicera diagrams originally published by Kaneko (1988).

| **Oribatid Mite Taxa** | **Abbreviated Name** | **δ^15^N Signature** | **δ^13^C Signature** | **Trophic Guild/ Feeding Guild and chelicera examples** |
| --- | --- | --- | --- | --- |
| *Ophidiotrichus tectus* | *O. tec.* | -8.39 | -25.99 | Phytophagous Feeders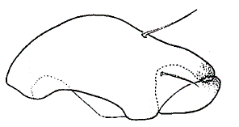 |
| *Parachipteria nicoleti* | *P. nic.* | -7.66 | -28.98 |  |
| *Hermannia convexa* | *H. con.* | -5.71 | -27.32 |  |
| *Carabodes areolatus* | *C. are.* | -5.03 | -28.97 |  |
|  | | | | |
| *Platynothrus peltifer* | *P. pel.* | -4.18 | -26.66 | Primary Decomposers/ Detritivorous feeders 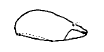 |
| *COMPOSITE LITTER/SOIL LAYER* | *NA* | -3.14 | -30.56 |  |
| *Ceratoppia quadridentata* | *C. qua.* | -2.66 | -28.62 |  |
| *Nothrus palustris* | *N. pal.* | -2.58 | -27.30 |  |
| *Chamobates pusillis* | *C. pus.* | -2.23 | -26.71 |  |
| *Euzetes globulus* | *E. glo.* | -1.73 | -30.19 |  |
| *Steganacarus magnus* | *S. mag.* | -0.94 | -28.06 |  |
|  | | | | |
| *Liebstadia similis* | *L. sim.* | -0.35 | -27.12 | Secondary Decomposers/ Fungivorous feeders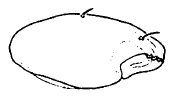 |
| *Phthiracarus italicus* | *P. ita.* | -0.32 | -21.76 |  |
| *Nanhermannia coronata* | *N. cor.* | -0.31 | -26.76 |  |
| *Nothrus silvestris* | *N. sil.* | -0.11 | -26.01 |  |
| *Rhysotritia duplicata* | *R. dup.* | 0.30 | -24.82 |  |
| *Acrogalumna longipluma* | *A. lon.* | 0.39 | -26.47 |  |
| *Phthiracarus anonymus* | *P. ano.* | 0.66 | -24.25 |  |
| *Oppiella (Moritzoppia) translamellata* | *O. tra.* | 0.84 | -28.17 |  |
| *Ceratozetes peritus* | *C. per.* | 1.24 | -26.69 |  |
|  | | | | |
| *Suctobelbella* spp. | *S. spp.* | 2.57 | -24.75 | Predators/Scavengers/Omnivorous feeders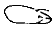 |
| *Oppiella (Rhinoppia) subpectinata* | *O. sub.* | 2.61 | -27.35 |  |
| *Hypochthonius rufulus* | *H. ruf.* | 2.74 | -26.24 |  |
| *Oppiella propinqua* | *O. pro.* | 2.85 | -27.51 |  |
| *Quadroppia ha?mmerae* | *Q. hem.* | 3.04 | -29.43 |  |
| *Quadroppia monstruosa* | *Q. mon.* | 4.78 | -29.28 |  |
| **Species not included in Stable Isotope Analysis** | | | | |
| *Ceratoppia sexpilosa* | NA | NA | NA | NA |
| *Dissorhina ornata* | NA | NA | NA |  |
| *Damaeus auratus* | NA | NA | NA |  |
| *Damaeus gracilipes* | NA | NA | NA |  |
| *Damaeobelba minutissima* | NA | NA | NA |  |
| *Eupelops acromios* | NA | NA | NA |  |
| *Liochthonius* spp. | NA | NA | NA |  |
| *Oribatella quadricornuta* | NA | NA | NA |  |
| *Pantelozetes paolii* | NA | NA | NA |  |
| *Punctoribates punctum* | NA | NA | NA |  |
| *Porobelba spinosa* | NA | NA | NA |  |
| *Tritegeus bisulcatus* | NA | NA | NA |  |

**Table S3** Table showing measured environmental factors per plot, number of oribatid trophic guilds (as defined by stable isotopes ^15^N/^14^N and ^13^C/^12^C) per plot and number of species occupying each trophic guild. Veg.: Vegetation Composition, Lit.: Litter density (high or low), Wat. Con.: Water content (%), Mean: average across six samples per plot, Std. Err: Standard Error across six samples per plot, No. of Guilds: number of guilds per plot and Phyto., Pri. Dec., Sec. Dec. and Pred.: Phytophagous, Primary Decomposer, Secondary Decomposer and Predatory trophic guilds, respectively.

| **Plot** | **Veg.** | **Lit.** | **Wat. Con.** | | **pH** | | **No. of Guilds** | **Number of Species** | | | |
| --- | --- | --- | --- | --- | --- | --- | --- | --- | --- | --- | --- |
|  |  |  |  |  |  |  |  | **Phyto.** | **Pri. Dec.** | **Sec. Dec.** | **Pred.** |
|  |  |  | **Mean** | **Std. Err.** | **Mean** | **Std. Err.** |  |  |  |  |  |
| 1 | Lu. | H | 64.901 | 1.605 | 3.883 | 0.137 | 4 | 2 | 6 | 8 | 5 |
| 2 | Lu. | L | 66.588 | 4.125 | 3.438 | 0.035 | 4 | 2 | 5 | 9 | 5 |
| 3 | Gr. | H | 45.526 | 7.928 | 3.760 | 0.136 | 4 | 3 | 6 | 9 | 5 |
| 4 | Gr. | L | 45.313 | 6.343 | 3.858 | 0.072 | 4 | 4 | 5 | 9 | 5 |
| 5 | Lu. | L | 44.536 | 4.320 | 4.468 | 0.268 | 4 | 3 | 6 | 9 | 5 |
| 6 | Lu. | H | 53.969 | 7.495 | 3.423 | 0.070 | 4 | 3 | 5 | 7 | 5 |
| 7 | Lu. | H | 48.417 | 6.785 | 3.835 | 0.092 | 4 | 2 | 5 | 9 | 5 |
| 8 | Gr. | L | 37.666 | 2.762 | 4.347 | 0.066 | 4 | 2 | 5 | 9 | 5 |
| 9 | Lu. | L | 47.188 | 6.011 | 3.997 | 0.053 | 4 | 3 | 4 | 7 | 5 |
| 10 | Gr. | H | 25.997 | 2.833 | 5.212 | 0.034 | 4 | 1 | 5 | 8 | 5 |
